# Both infected and uninfected mosquitoes are attracted toward malaria infected birds

**DOI:** 10.1186/1475-2875-12-179

**Published:** 2013-06-03

**Authors:** Stéphane Cornet, Antoine Nicot, Ana Rivero, Sylvain Gandon

**Affiliations:** 1 Centre d’Ecologie Fonctionnelle et Evolutive (CEFE), UMR CNRS 5175, Montpellier, France; 2 Maladies Infectieuses et Vecteurs: Ecologie, Génétique, Evolution et Contrôle (MIVEGEC), UMR CNRS 5290-IRD 224-UM1-UM2, Institut de Recherche pour le Développement, 911 av. Agropolis, Montpellier 34394, France

**Keywords:** *Plasmodium relictum*, Malaria, Vector behaviour, Attractiveness

## Abstract

**Background:**

The biting behaviour of mosquitoes is crucial for the transmission of malaria parasites. This study focuses on the feeding behaviour of *Culex pipiens* mosquitoes with regard to the infection status by the avian malaria parasite *Plasmodium relictum* (lineage SGS1).

**Methods:**

Uninfected and sporozoite-infected mosquitoes were provided with a choice between an uninfected bird and a bird undergoing a chronic *P. relictum* infection. Mosquito choice is assessed by microsatellite typing of the ingested blood.

**Results:**

Chronically infected birds are more attractive to mosquitoes. This choice is not altered by the infection status of the mosquitoes: both infected and uninfected mosquitoes have similar host choice behaviours and are more attracted towards infected birds.

**Conclusions:**

These results support some, but not all predictions derived from the hypothesis that malaria parasites can manipulate the behaviour of their mosquito vectors to enhance their transmission. The possible mechanisms driving this manipulation, the evolutionary dynamics leading to the modification of the biting behaviour of mosquitoes by *Plasmodium sp.* as well as the implications for malaria epidemiology are discussed.

## Background

The manipulation of host phenotypes by parasites, aiming at enhancing parasite transmission, is a widespread phenomenon having far-reaching ecological and epidemiological implications [1,2]. In vector-borne diseases the completion of pathogen transmission requires: (i) uninfected vectors to bite infected hosts; and, (ii) infected vectors to bite uninfected hosts. There is growing evidence that parasites may manipulate their vectors at both of these stages of its life cycle.

Parasites have developed strategies to modify the behaviour of uninfected vectors. There is, indeed, growing evidence that uninfected vectors show a preference for feeding on infected hosts [3-7]. Such a preference has been shown in uninfected mosquitoes faced with both human [8] and avian [9]*Plasmodium sp.* infections. Using the avian malaria parasite *Plasmodium relictum* (lineage SGS1), Cornet *et al.* have recently shown that malaria infection increases the attractiveness of domestic canaries to uninfected *Culex pipiens* mosquitoes, one of the main natural vectors of this parasite [9]. Despite the fact that taking an infected blood meal is costly for the vector (it results in a 30% reduction of mosquito fecundity, [10]) uninfected mosquitoes showed a clear preference towards biting chronically infected birds [9]. A likely adaptive explanation for this seemingly paradoxical behaviour from the mosquitoes’ point of view is that it is the result of parasite manipulation. Although the proximal cause leading to the increased attractiveness of infected birds is as yet unclear, it is likely to involve the modification of key odorant volatiles [4,9].

Infected vectors may also be manipulated by the parasite and in different ways. Vectors infected with transmissible parasite stages of *Plasmodium*[11-18], *Leishmania*[19,20] and dengue virus infection [21] have been shown to exhibit increased attraction to hosts, higher host probing/biting rates and/or longer blood meals. In addition, infected vectors are also more likely to feed on multiple hosts [20,22]. A high-bite strategy, which is probably achieved through modifications in the composition of the saliva that hamper the blood meal process [12,23,24], can be particularly advantageous for parasite transmission, specially if some of the extra bites take place in new, uninfected hosts. Another way to increase transmission may involve the modification of the host choice behaviour of the infected vector. Parasite transmission could be increased if infected vectors avoid within-host competition by feeding preferentially on uninfected hosts. Within-host competition can take different forms [25]. Parasites may compete directly for host resources, such as a particular cell or tissue, or a key limiting nutrient in short supply (exploitation competition). In addition, parasites may compete indirectly via the non-specific arm of the host’s immune system (immune-mediated apparent competition). Teasing apart which mechanism is at play is not an easy task, but there is evidence from malaria that both of these mechanisms may be acting simultaneously [26-31]. The study of the host-choice behaviour of infected vectors remains, however, poorly studied. Some infected vectors of plant pathogens have been shown to be manipulated towards uninfected plants [5,6]. To date, no studies have ever looked at the host choice behaviour of malaria-infected mosquitoes.

The present study investigates the biting behaviour of both uninfected and sporozoite-infected *Cx. pipiens* mosquitoes when given the choice between birds that are uninfected or in chronic *P. relictum* infection. The number of mosquitoes that fed on each host has been quantified using genetic (microsatellite) analyses of the blood meal [9]. This system gives a more biologically relevant measure of host choice as it captures the whole behavioural sequence from the detection of the odour to the decision to bite.

This experimental set up allowed testing three predictions deriving from the manipulation hypothesis. First, uninfected mosquitoes are expected to feed preferentially on the infected birds [9]. Second if, as discussed above, *P. relictum* can manipulate the biting rate of the infected mosquitoes, one may expect the infected mosquitoes to have a higher probability of feeding and a higher probability of multiple-host biting than uninfected mosquitoes. Third, if *P. relictum* can manipulate the host-choice of the infected mosquitoes, one may expect the vectors to avoid within-host competition by preferentially biting the uninfected hosts. Note that this may generate a conflict of interest between the parasite in the bird (trying the attract mosquitoes) and the parasite in the mosquito (trying to avoid infected birds). The experiment is designed to study how this conflict is resolved and to obtain a more precise description of malaria transmission. As discussed below, a better knowledge of the biting behaviour of the vector can have important consequences for the epidemiological dynamics of vector-borne diseases [32,33].

## Methods

### Malaria parasites

*Plasmodium relictum* (lineage SGS1) is the aetiological agent of the most prevalent form of avian malaria in Europe [34] and is highly prevalent in wild passerines [35-37]. This generalist *P. relictum* parasite lineage was originally isolated from wild house sparrows caught in the region of Dijon (France) in 2009 [38] and maintained in the laboratory via subsequent passages to naïve canaries by intraperitoneal injection or by completing the parasite cycle through mosquitoes. Mosquitoes of the *Cx. pipiens* complex are the main vectors of *P. relictum* in the field [37].

### Infected and uninfected mosquitoes

Experiments were conducted with a laboratory strain of *Cx. pipiens quinquefasciatus* (SLAB) [39]. Mosquitoes were reared under standard conditions [40]. Infected and uninfected mosquitoes for the experiment were obtained in the following way. Eight cages (dimensions L40 × W30 × H30 cm), each containing 150 female (six to seven days old) mosquitoes were set up. Half of these cages were provided with an infected canary, the other half with an uninfected one. Infected birds had been inoculated with the parasite 12 days previously following standard laboratory procedures, and were thus at the acute stage of the infection. Previous work has shown that this protocol ensures that >90% of the mosquitoes become infected [40]. Unfed mosquitoes were discarded. Four days after the infected or uninfected blood meal (day 4 pbm), and until the beginning of the behavioural assay, mosquito cages were provided with a water-filled plastic tray to allow females to lay their eggs. On day 7 pbm, a subsample of ten mosquitoes were haphazardly collected from each of the infected cages and dissected to verify the presence of oocysts in the midgut [40]. Only the three of the four infected cages that reached a prevalence of infection of 100% were kept for the experiment. A similar sample of mosquitoes was collected from the uninfected cages to verify the absence of parasite.

Once mature (eight to nine days pbm), oocysts burst and release sporozoites into the mosquito body cavity that rapidly colonize the salivary glands within hours [41]. Parasite sporozoites can be detected within the glands starting from day 10 pbm (A Nicot, unpublished). The timing of the behavioural assay was set to coincide with the peak of sporozoite presence in the salivary glands of infected mosquitoes (12–14 days pbm). Given that *P. relictum* sporogony is asynchronous, some midgut oocysts may, however, still be developing at this stage (see [42]). Four days before the behavioural assays, mosquitoes were marked using small amount of either pink or yellow fluorescent powder (RadGlo_JST, Radiant Color NV, Houthalen, Belgium) applied as a dust storm [40]. The two colours were used in rotation to mark uninfected and infected mosquitoes.

### Infected and uninfected birds

One year-old canaries (*Serinus canaria*) were used in this experiment. A small amount (*ca.* 20–30 μL) of blood was collected from the brachial vein and used for molecular sexing [43] and genotyping at the microsatellite Cuμ28 locus [9,44]. At the same time, the birds were verified to be free from any haemosporidian infections [45]. Birds were then assorted into ten different same-sex pairs making sure that birds within a pair had different microsatellite profiles at the Cuμ28 locus [9]. Within each pair, one bird was randomly chosen to be infected. Infections were carried out using an intraperitoneal injection of *ca.* 50–100 μL of blood from the infected canary stock [9]. The success of the infection was verified 11 days post-infection (dpi) using thin blood smears stained with Giemsa.

### Behavioural assay

The behavioural assay took place 53–55 dpi, to coincide with the chronic stage of the bird infection. On the same day, but prior to the assay, a small amount (*ca.* 20–30 μL) of blood was taken from the brachial vein of each of the birds to measure parasitaemia and haematocrit level. Parasitaemia was established using previously published qPCR procedures [9]. Haematocrit level was expressed as the packed cell volume (PCV) volume of red blood cells per total volume of blood in the capillary after centrifuging blood for 5 min at 10,000 rpm [9].

To minimize host defensive behaviours that may alter the mosquito feeding process during the assay [46], birds were immobilized in a specially designed PVC tube that rendered their legs accessible to the mosquitoes while protecting the rest of the body from the bites [9]. Each bird pair was placed inside a cage (dimensions L80 × W30 × H30 cm) with 40 uninfected and 40 sporozoite-infected female mosquitoes for two hours (from 6 to 8 pm). Each batch of 40 infected and 40 uninfected mosquitoes contained an equal proportion of mosquitoes from the three infected and four uninfected cages. To avoid interference, each pair of birds was assessed in a separate controlled temperature room (four different CT rooms, temperature: 25 ± 1°C, relative humidity: 73 ± 3%). The experiment was spread over three consecutive evenings. After each run, all mosquitoes were taken out of the cage and stored at −80°C for microsatellite analyses. Microsatellite analyses of the blood fed mosquitoes were carried out using a previously published protocol [9]. Mosquitoes were sorted as unfed when no microsatellite signal was detected.

### Statistics

The analyses were carried out using generalized linear mixed models (glmer, package lme4) available in the R statistical package (v. 2.15.2). The binomially distributed response variables were: the proportion of mosquitoes that took a blood meal (blood feeding success), the proportion of mosquitoes that fed on both birds (multihost biting), and the proportion of mosquitoes that bit the infected bird relative to the total number of blood-fed mosquitoes (multihost feeders were eliminated from this analysis). Models were fitted by specifying mosquito infection treatment (infected, uninfected) as a fixed effect and the bird pair identity as a random effect [47]. In addition, the effect of ∆ haematocrit (the difference in haematocrit between the infected and the uninfected bird) and blood parasitaemia on vector-feeding preference were investigated [9]. Maximal models were simplified by sequentially eliminating non-significant terms and interactions (*p* > 0.05) and comparing the change in deviance with and without the term using a *χ*^2^ distribution. The effect of *P*. *relictum* infection on bird attractiveness was confirmed by using a replicated *G*-test of goodness of fit [9,48]. The observed attractiveness of the infected bird (proportion of mosquitoes biting the infected bird) was tested against the predicted no-choice value of *p* = 0.5 (half of the mosquitoes bite each of the birds).

### Ethical statement

Animal experiments were carried out in strict accordance with the “National Charter on the Ethics of Animal Experimentation” of the French Government, and all efforts were made to minimize suffering. Experiments were approved by the Ethical Committee for Animal Experimentation established by the authors’ institution (CNRS) under the auspices of the French Ministry of Education and Research (permit number CEEA- LR-1051).

## Results

The status of birds was confirmed by qPCR assays: control birds were parasite-free and experimentally infected birds were all positive for *P. relictum* infection. Since birds harboured chronic (low intensity) infections, the infection status did not affect bird haematocrit (mean ± se: uninfected 0.43 ± 0.02, infected 0.40 ± 0.03, *F*_1, 18_ = 0.93, *p* = 0.3485) and overall, within pairs, uninfected and infected birds had similar haematocrit values (one-sample *t*-test *t*_1,9_ = −1.16, *p* = 0.2763).

**Figure 1:**
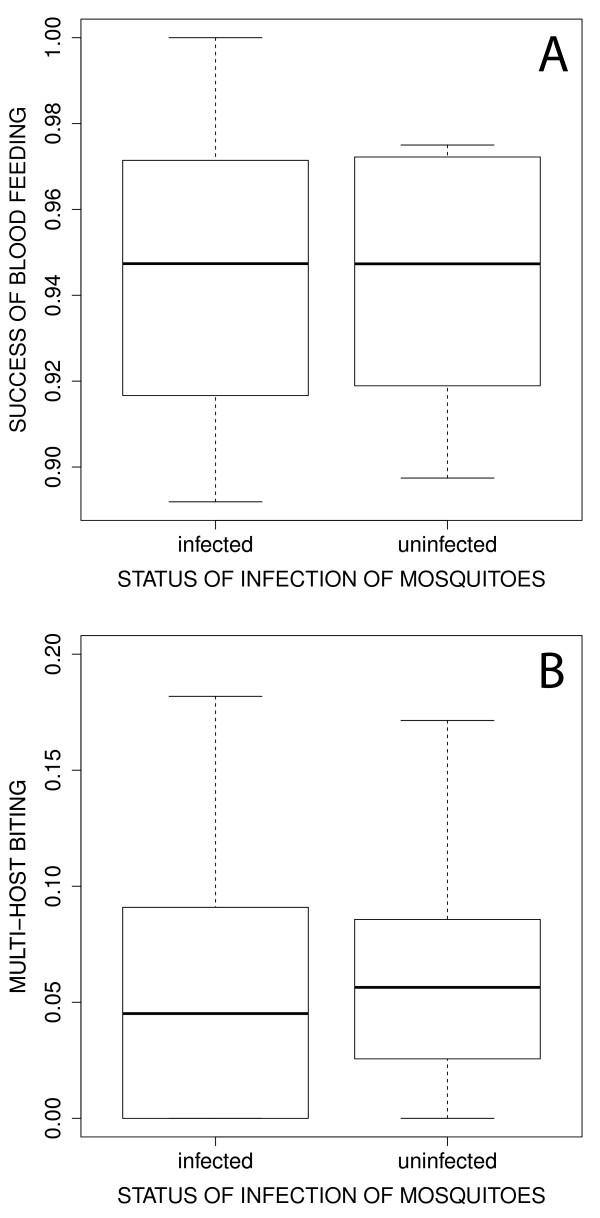
**Proportion of blood-fed mosquitoes retrieved after the behavioural assays according to the infection status of mosquitoes (sporozoite-infected *vs *uninfected by *Plasmodium relictum*).** (**A**) Total blood feeding success. (**B**) Multihost biting. Boxes are interquartile ranges, thick lines are medians and bars enclose 90% of the distribution.

The majority of mosquitoes (> 90%) took a blood meal and the success of engorgement did not depend on whether mosquitoes were infected (mean ± se: 0.945 ± 0.010) or uninfected (0.942 ± 0.009, χ^2^_1_ = 0.05, *p* = 0.8261, Figure 1A). The proportion of mosquitoes that fed on both birds was low and did not differ between infected (mean ± se: 0.061 ± 0.019) and uninfected mosquitoes (0.062 ± 0.016, χ^2^_1_ = 0.007, *p* = 0.9424, Figure 1B).

**Figure 2:**
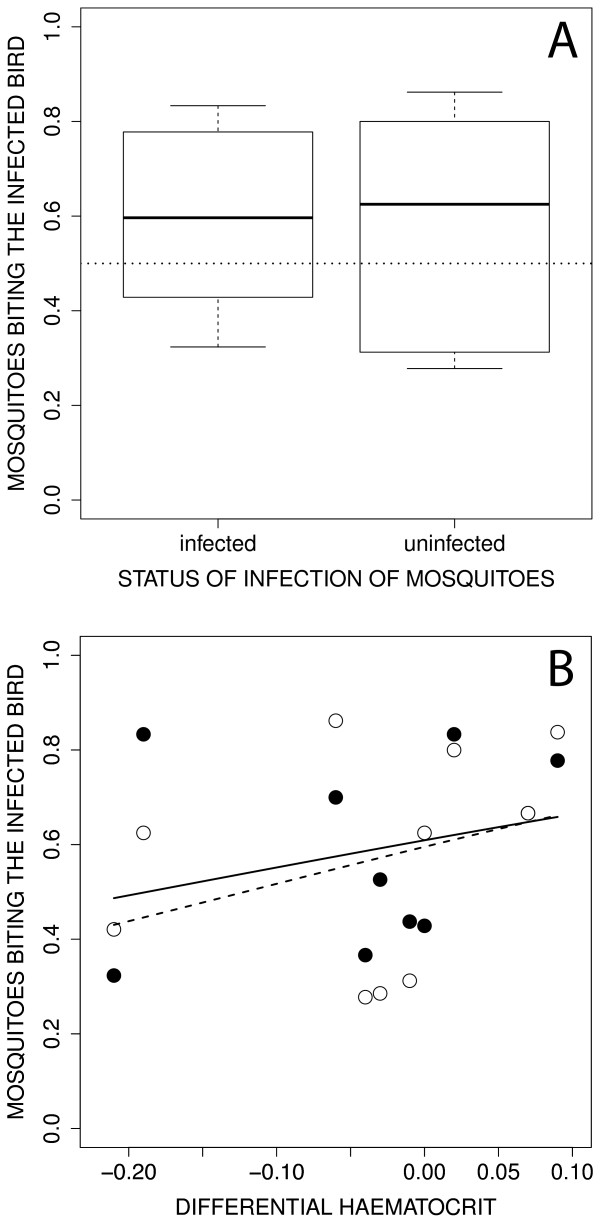
**Attractiveness of canaries infected by *Plasmodium relictum *to *Culex pipiens *mosquitoes according to their status of infection by *Plasmodium relictum *(infected by sporozoites, uninfected).** Bird attractiveness refers to the proportion of mosquitoes that bit the infected bird relative to the number of blood-fed mosquitoes. (**A**) Raw data. Boxes are interquartile ranges, thick lines are medians and bars enclose 90% of the distribution. The dotted line represents the proportion in the absence of choice (*p* = 0.5). (**B**) Relationships between bird attractiveness of the infected bird and differential haematocrit, which refers to the difference in haematocrit between the infected bird and the uninfected control bird within pairs. Uninfected mosquitoes: open circle, dashed line; sporozoite-infected mosquitoes: dark circle, full line. Lines are the fits of GLM models.

The global GLMM highlighted that the infection status of mosquitoes did not affect the feeding preference (χ^2^_1_ = 0.29, *p* = 0.5901; Figure 2A). There was neither an effect of ∆ haematocrit (χ^2^_1_ = 0.97, *p* = 0.3252) or bird parasitaemia (χ^2^_1_ = 0.03, *p* = 0.8670) on vector choice. Both uninfected and sporozoite-infected mosquitoes preferred feeding on infected birds, however, in this model, this preference was not significant given the model intercept did not represent a significant deviation from 0.5 (*p* = 0.0985). Given the absence of an effect of mosquito infection on feeding choice, and since there is still a debate about the correct choice of degrees of freedom and the robustness of GLMM analyses, a simplified GLM was run to investigate bird attractiveness using all mosquitoes, independently of their infection status, thus discarding the random term effect (see below).

**Figure 3:**
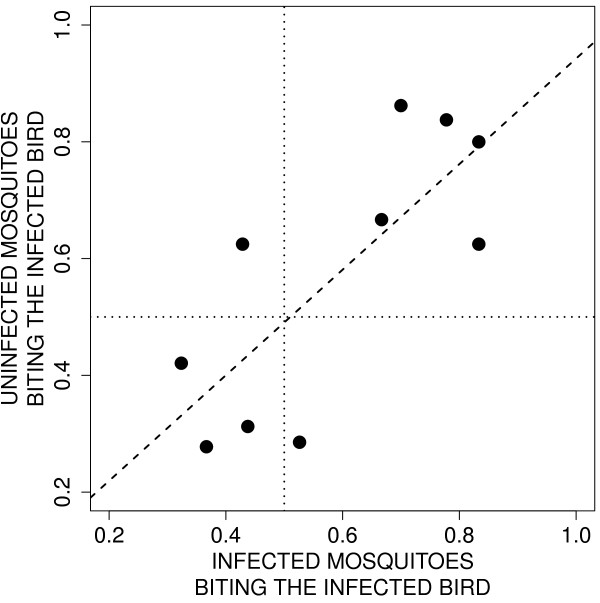
**Covariation between the proportion of uninfected *vs. *sporozoite-infected mosquitoes biting the bird infected with *Plasmodium relictum. *** The significant positive relationship (dash line) highlights that, independently of the intrinsic attractiveness of the infected bird, infected and uninfected mosquitoes had a similar host-choice preference. The dotted lines represent the proportion in the absence of choice (*p* = 0.5).

Infected birds attracted significantly more mosquitoes (60.3% of the mosquitoes) than uninfected birds (χ^2^_9_ = 104.05, *p* < 0.0001; Figure 2A). A replicated *G*-test of goodness of fit confirmed a statistically significant departure from the frequency in the absence of choice (p = 0.5) (total-*G* = 106.67, 10 *df*, *P* < 0.0001; pooled-*G* = 15.16, 1 *df*, *P* < 0.0001), albeit with a significant heterogeneity between the replicates (heterogeneity-*G* = 91.50, 9 *df*, *P* < 0.0001). There was a significant positive effect of ∆ haematocrit on the proportion of mosquitoes biting the infected bird (Figure 2B; χ^2^_1_ = 11.05, *p* = 0.0009), suggesting that mosquitoes prefer biting the birds with high haematocrit values. However, bird parasitaemia did not quantitatively influence the strength of mosquito feeding behaviour (χ^2^_1_ = 0.25, *p* = 0.6161). Further analyses confirmed that infected and uninfected mosquitoes behaved in the same way: within each bird pair there was a significant positive covariation in the choice of infected and uninfected mosquitoes (*r* = 0.767, *F*_1, 8_ = 11.45, *p* = 0.0096; Figure 3).

## Discussion

A previous experiment has shown that *P. relictum* infection increases bird attractiveness to mosquitoes: about 60% of uninfected mosquitoes preferred feeding on birds with a chronic infection (24–26 dpi) compared to uninfected controls [9]. In the present study, results are quantitatively similar, albeit in even older infections (53–55 dpi). The haematocrit effect found in the previous paper is also present here: mosquitoes prefer biting the least anaemic birds. Avian malaria infections can last for months in their chronic state (S Cornet *et al.*, unpublished; [49]). An increase in the attractiveness of the birds during this stage may thus enhance considerably malaria transmission. This result is thus consistent with the first prediction of the manipulation hypothesis.

In contrast, the second of the predictions, concerning the probability of biting of infected mosquitoes does not seem to be supported by the data. There was no difference between uninfected and sporozoite-infected *Cx. pipiens* in either the blood feeding success (Figure 1A) or the rate of multihost biting (Figure 1B). This contrasts with earlier studies reporting an increased attraction to hosts [17,18] and altered biting rate of *Plasmodium sp.*-infected mosquitoes [12-16,22]. Here, however, it was impossible to control for the duration and the volume of the blood meals. More detailed behavioural studies should be carried out on avian malaria to investigate the possibility that *P. relictum* manipulates these components of the dynamics of the biting behaviour of infected mosquitoes.

Finally, contrary to the third and final prediction of the manipulation hypothesis, uninfected and sporozoite-infected mosquitoes behaved in a similar way (Figure 3): they were both preferentially attracted by chronically infected birds (Figure 2). Biting an infected host was predicted to be costly for the malaria parasite within the mosquito because of within-host competition with resident parasites and/or immune mediated apparent competition [25]. In several plant pathogen systems, infected vectors are indeed preferentially attracted toward uninfected plants [5,6]. However, the results show that, in the avian malaria system, what is driving the host-choice of infected mosquitoes is the malaria parasite within the bird, not the malaria parasite within the mosquito. In this experiment, the parasite in the bird and in the mosquito came from the same parasite stock (lineage SGS1), differing only by one serial passage. This brings up the question of whether the results would have been significant if two different parasite stocks, or parasite lineages, had been used to infect the birds and the mosquitoes. The answer is likely to be no, because costs for the mosquito-inhabiting parasite of biting an already infected host are maximal when the bird-residing parasite is a relative. An analogous problem is found in parasitoid wasps that encounter hosts that have been already parasitized, either by itself or by a different female. In these circumstances, females have been shown to avoid self-superparasitism and to prefer hosts parasitized by conspecifics to avoid the costs of sibling competition [50].

What is driving the bias in the host-choice behaviour in mosquitoes? Plant-pathogen studies have gone very far in deciphering the underlying cues used by the vectors to choose between infected and uninfected plants. Some of these cues involve the colour, the quality and the odours of the plants [4-6]. For malaria, the vectors often bite in the dark and the choice is probably not guided by visual cues. The quality of the blood meal is likely to play a role on this choice: mosquitoes are consistently attracted towards birds with a high haematocrit (see also [9]). High haematocrit correlates with a higher protein content [51] which may, in turn, correlate with a higher mosquito fecundity or longevity although, no study to date has investigated the correlation between anaemia in the host and mosquito fitness. Yet, whether and, if so, how do mosquitoes evaluate the haematocrit of the birds is an open question. Given that many diseases are associated with changes in host odour profile [52], it is likely that odour volatiles provide a lot of information to the vectors. Body odours emanating from the gland secretions and the skin microflora play an important role in mosquito attraction in humans [18,53,54]. In the *P. relictum – Cx. pipiens* – *S. canaria* system, the quantity and the composition of these volatiles may provide information to the mosquitoes about the infection status and haematocrit levels of the bird. A bird-derived odorant molecule, the nonanal, has recently been identified to be a strong attractant for *Culex* mosquitoes [55] and could potentially be a key molecule in this system.

Understanding the proximal cues of the attraction may help understand the evolution of the mosquito manipulation by the malaria parasite. For example, bird infection may act on the quantity but not on the composition of the odour. In this case, the increased preference for infected birds would be triggered by the exaggeration of a pre-existing cue or a cue that is already used by mosquitoes to locate hosts [4]. Evolutionary speaking, such deceptive signals are a good way to prevent the evolution of resistance against the attractant in the vector population, because, for both infected and uninfected mosquitoes, the fitness costs of ignoring such signals are large (no blood meal means no reproduction). This shows that the next experimental step in the study of mosquito behaviour involves deciphering the odours emitted by the birds and relating these to mosquito choice.

Mosquito choice may have a huge impact on the epidemiology of malaria transmission. In particular, Kingsolver [32] showed that the higher attractiveness of infected hosts increases the basic reproductive number, *R*_0_, of malaria. In other words, the effect reported in the present study could increase the initial spread of the epidemic. One interesting situation may take place when parasite prevalence is high. In this case, increasing the attractiveness of infected hosts may protect uninfected hosts, eventually decreasing the incidence of malaria in the vertebrate population. In this particular situation, and contrary to the adaptive hypothesis presented throughout this paper, a preference for infected hosts may not be adaptive for the malaria parasite. To investigate the adaptive nature of these phenotypic alterations an evolutionary model is currently being developed where the choice between infected and uninfected hosts will be allowed to evolve under different scenarios depending on who is controlling the phenotype: the vector, the parasite in the host or the parasite in the infected vector. This may help understand the evolution of malaria but also the transmission of many other vector-borne diseases.

## Conclusion

This study is the first to investigate the combined effect of infection by malaria parasites on host attractiveness and vector feeding preference using a behavioural choice experiment over the whole *P. relictum* life cycle. Both sporozoite-infected and uninfected mosquitoes have similar biting behaviours and are more attracted towards birds in the chronic infection stage. These results support some, but not all predictions derived from the hypothesis that malaria parasites can manipulate the behaviour of their mosquito vectors to enhance their transmission. Although the importance of manipulation by malaria parasites for disease transmission in the field remains to be confirmed [56], the avian malaria system provides an unparalleled opportunity to study these questions in depth. The results obtained using this animal model, which associates a natural vector-*Plasmodium sp.* combination, will help improve our understanding of malaria epidemiology.

## Competing interests

The authors declare that they have no competing interests.

## Authors’ contributions

SC, AR and SG conceived and designed the experiment. SC performed the experiment and analysed the data. AN performed the molecular analyses, SC, AR and SG wrote the paper. All authors read and approved the final manuscript.
